# Accuracy of preoperative MRI to assess lateral neck metastases in papillary thyroid carcinoma

**DOI:** 10.1007/s00405-017-4728-z

**Published:** 2017-09-02

**Authors:** Suvi Renkonen, Riikka Lindén, Leif Bäck, Robert Silén, Hanna Mäenpää, Laura Tapiovaara, Katri Aro

**Affiliations:** 1 Department of Otorhinolaryngology, Head and Neck Surgery, University of Helsinki and Helsinki University Hospital, P.O. Box 263, 00029 Helsinki, Finland; 2 Department of Biosciences and Nutrition, Karolinska Institutet, Stockholm, Sweden; 3 Department of Radiology, HUS Medical Imaging Centre, University of Helsinki and Helsinki University Hospital, Helsinki, Finland; 4 Faculty of Medicine, University of Helsinki, Helsinki, Finland; 5 Department of Oncology, Nuclear Medicine, Cancer Center, University of Helsinki and Helsinki University Hospital, Helsinki, Finland; 6 Department of Dentistry, University of California, Los Angeles, Los Angeles, CA USA

**Keywords:** Papillary thyroid carcinoma, Lateral neck metastasis, MRI, Neck dissection, Negative predictive value

## Abstract

Primary treatment of papillary thyroid carcinoma (PTC) with lateral lymph node metastasis is surgery, but the extent of lateral neck dissection remains undefined. Preoperative imaging is used to guide the extent of surgery, although its sensitivity and specificity for defining the number and level of affected lymph nodes on the lateral neck is relatively modest. Our aim was to assess the role of preoperative magnetic resonance imaging (MRI) in predicting the requisite levels of neck dissection in patients with regionally metastatic PTC, with a focus on Levels II and V. All patients with PTC and lateral neck metastasis who had undergone neck dissection at the Department of Otorhinolaryngology—Head and Neck Surgery, Helsinki University Hospital, Helsinki, Finland from 2013 to 2016 and had a preoperative MRI available were retrospectively reviewed. A head and neck radiologist re-evaluated all MRIs, and the imaging findings were compared with histopathology after neck dissection. In the cohort of 39 patients, preoperative MRI showed concordance with histopathology for Levels II and V as follows: sensitivity of 94 and 67%, specificity of 20 and 91%, positive predictive value of 56 and 75%, and negative predictive value of 75 and 87%, respectively. In PTC, MRI demonstrated fairly high specificity and negative predictive value for Level V metastasis, and future studies are needed to verify our results to omit prophylactic dissection of this level. Routine dissection of Level II in patients with regionally metastatic PTC needs to be considered, as MRI showed low specificity.

## Introduction

Papillary thyroid carcinoma (PTC) accounts for approximately 75% of all thyroid malignancies and more than 90% of differentiated thyroid carcinomas [1, 2]. Primary treatment of PTC consists of surgery, typically total thyroidectomy with or without central lymph node dissection, adapted radioiodine ablation and postoperative TSH suppression [1, 3, 4]. PTC carries an excellent prognosis due to the slow growth and minimally invasive behavior with survival rates over 90% [5–7]. However, lymph node metastasis (LNM) in PTC is relatively common, as seen in 50–90% of patients [1, 3, 5, 8]. LNMs impact overall patient survival especially in patients over 45 years of age [9, 10]. Furthermore, LNMs in the lateral neck seem to increase the risk for locoregional recurrences and distant metastases [8], emphasizing the importance of detailed preoperative evaluation in the primary treatment setting.

High-resolution ultrasound (US) and magnetic resonance imaging (MRI) are valuable in preoperative diagnostics of thyroid carcinoma [2, 11]. US is sensitive in the evaluation of the neck, and with the possibility of simultaneous needle biopsies [11]. However, US has suboptimal accuracy in assessing the levels of LNMs, especially for Levels II and V [12]. In comparison with US, MRI is superior especially in assessing extensive or bulky neck lymphadenopathy in the mediastinum or paratracheal, parapharyngeal, and retropharyngeal regions when experience in interpreting US is insufficient [13]. However, in the most recent guidelines from the American Thyroid Association (2015), only US is recommended for the routine evaluation of the neck prior surgery for differentiated thyroid carcinomas [13].

Although comprehensive neck dissection (ND) seems to decrease recurrences on the neck, the extent of ND needed in patients with PTC with LNM remains unclear [8, 14]. As current data on the role of MRI in demonstrating the levels of lateral neck metastases are limited, we assessed correlation of MRI findings with histopathology, focusing on the accuracy of preoperative MRI in determining the requisite ND levels.

## Materials and methods

All patients, who underwent lateral ND for preoperatively cytologically and/or radiologically confirmed LNM of PTC at the Department of Otorhinolaryngology—Head and Neck Surgery, Helsinki University Hospital, Helsinki, Finland, from January, 2013 to February, 2016, were included in the study. The referral area comprises a population of 1.6 million. Only patients with a diagnostic preoperative MRI and without any previous lateral neck operation were included. At our institution, we prefer MRI instead of computed tomography (CT) in this patient population to avoid radiation exposure and to show possible increased T1 signal intensity.

Preoperative MRI was often used to help in defining the levels to be included in lateral ND. Still, surgeon’s personal views and patient characteristics influenced the extent of ND. In addition, the initial MRI reports often lacked specific information on the suspicious levels and number of lateral neck metastases, as well as of the possible extranodal extension (ENE). Thus, to standardize the reports, all studies were re-evaluated by an experienced head and neck radiologist (R.L.), who assessed the affected levels, and estimated the number of LNMs. These two reports were compared to show any interobserver difference. The imaging data were compared level-by-level with histopathology from the ND preparate to determine their correlation. US was performed either in the referring institution, or at our institution (*n* = 27, 69%), and US data were also compared with histopathology level-by-level, when possible. Postoperative complications were classified according to the Clavien–Dindo classification of surgical complications [15].

## MRI

The MRI scans were performed on a 1.5 T (*n* = 32) or a 3 T (*n* = 7) MRI scanner with a neck coil in different imaging centres in the referral area using the following pulse sequences: axial fat-suppressed T2-weighted images, axial T1-weighted images, and gadolinium-enhanced fat-suppressed axial and coronal T1-weighted images. Slice thickness was 3 or 4 mm. These imaging sequences were required to be regarded as a high-quality and diagnostic MRI. In addition, axial fat-suppressed T1-weighted pre-gadolinium images were acquired in 33 patients to illustrate the possible increased T1 signal intensity.

**Fig. 1:**
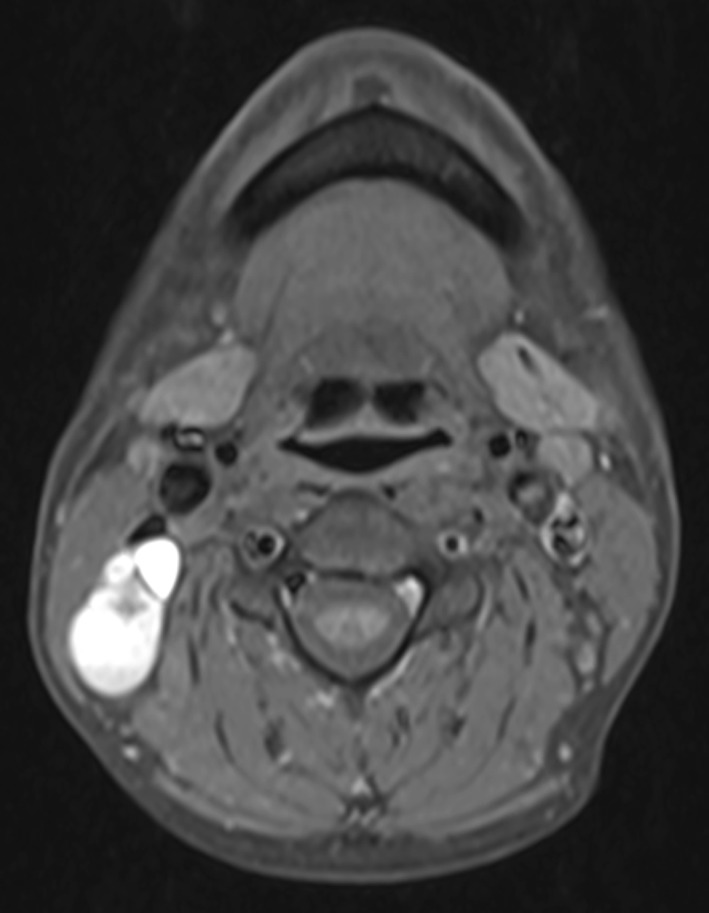
Fused, metastatic T1 hyperintense Level II lymph nodes. The minimal diameter of the largest node is 16 mm. Contrary to CT or US, MRI can identify increased T1 signal intensity indicating thyroglobulin

**Fig. 2:**
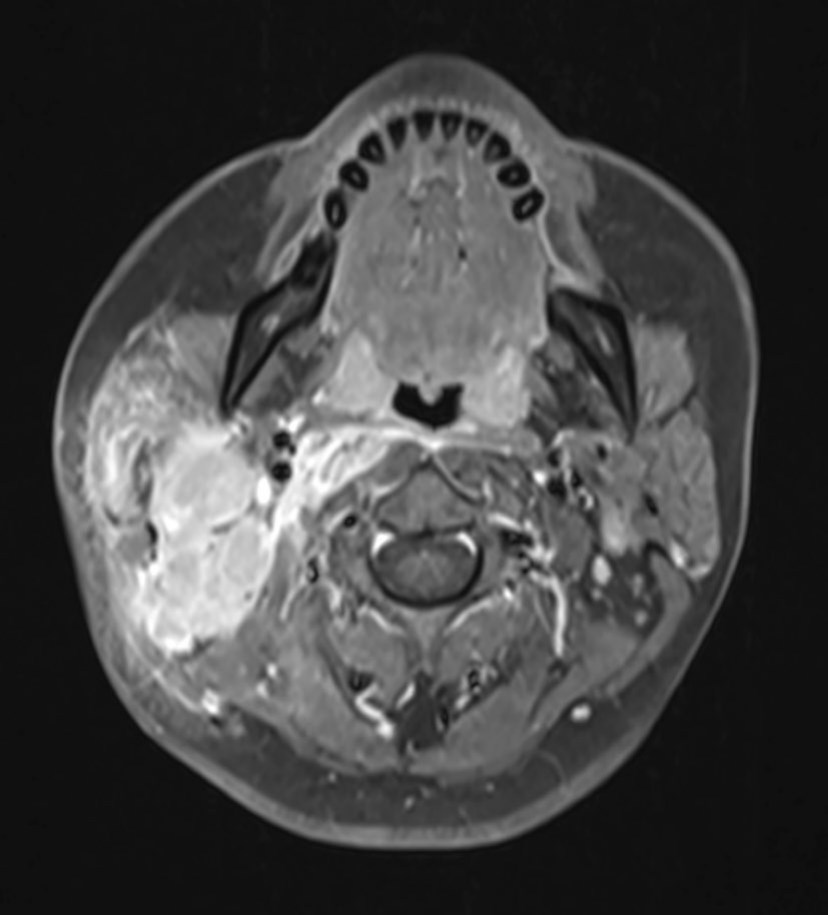
Gadolinium-enhanced T1 weighted fat-saturated image of Level II fused nodes. The nodes enhance unevenly indicating central necrosis and show enhanced edema in the surrounding soft tissue indicating extranodal extension

**Fig. 3:**
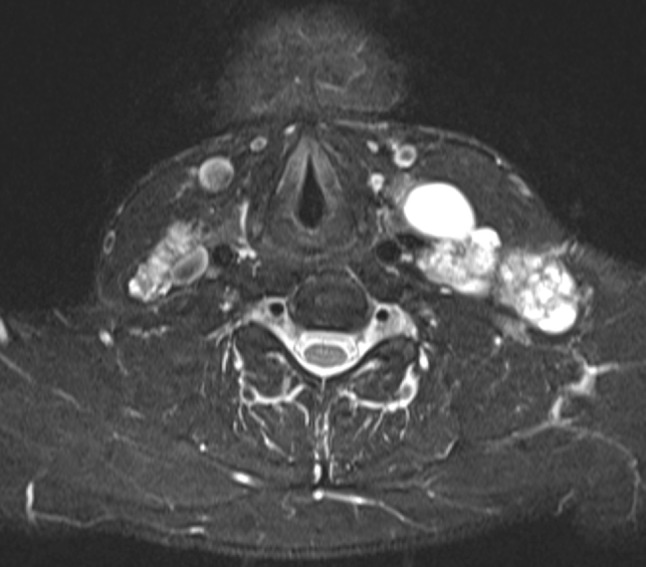
T2 fat-saturated image of bilateral fused metastatic lymph nodes. *On the left* the larger lymph node aggregate exhibits multifocal cystic change and extends to Levels II, III, IV, and V

**Fig. 4:**
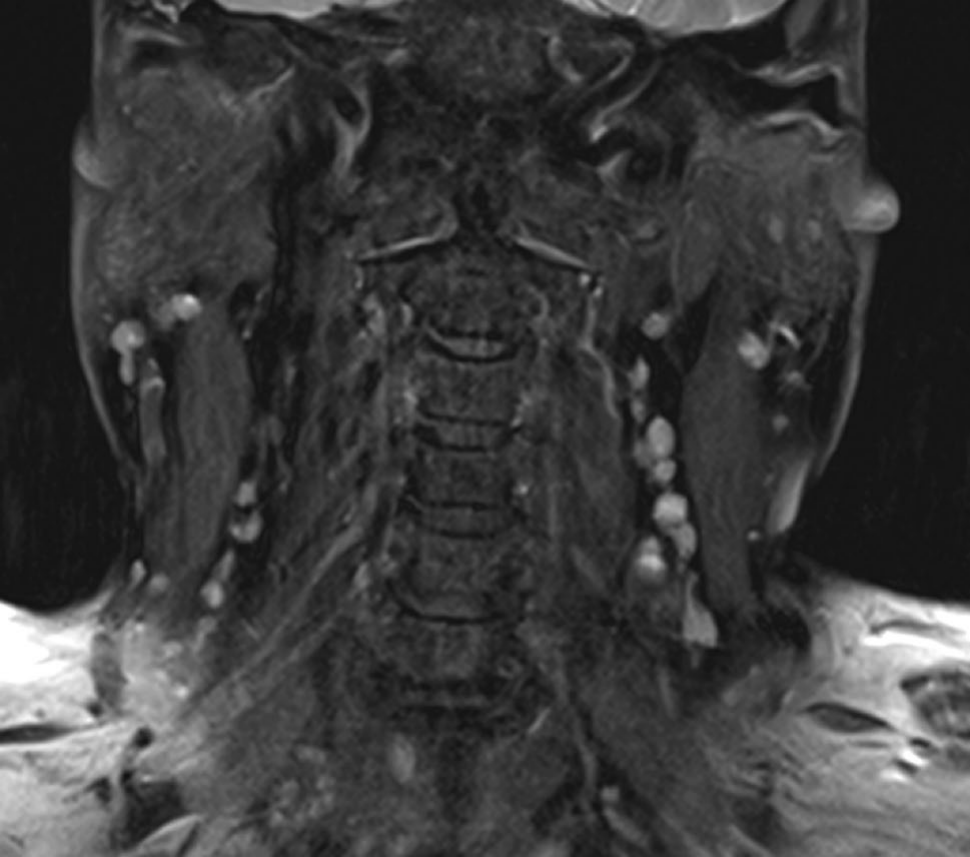
Fat-saturated T2 coronal image with several small and clustered lymph nodes in Level III *on the left*; some of the nodes are round in shape

A head and neck radiologist (R.L.) reviewed the MRI images in a blinded fashion, without knowledge of the specific histopathology from ND. A node was considered malignant if it demonstrated increased T1 signal intensity (Fig. 1) or if: (1) the minimal diameter of the node exceeded 10 mm (11 mm for digastric nodes); or (2) it showed central necrosis (Fig. 2), ENE (Fig. 2) or cystic change (Fig. 3); or (3) it was round in shape (Fig. 4); or (4) nodes were fused (Figs. 1, 2, 3). In addition, small, clustered nodes were considered suspicious and rated as malignant (Fig. 4). A lymph node that involved two adjacent levels was rated to situate in the level that encompassed the largest volume. In the re-evaluation, criteria for ENE were: (1) presence of high-intensity signal in the tissues around the node on fat-suppressed T2-weighted images or (2) an irregular nodal margin.

## Results

The study cohort consists of 39 patients operated for lateral LNM of PTC at our institution, and the baseline demographics are presented in Table 1. Re-evaluation of MRI scans did not change the estimate of the general distribution of LNMs.

**Table 1:** Baseline demographics of 39 patients with papillary thyroid carcinoma

Age, mean	45.5 years (range 18–82)
Gender; *n* (%)	Women 23 (59) Men 16 (41)
Previous cancer; *n* (%)	No 34 (87) Yes 5 (13)
Preoperative ultrasound at our institution; *n* (%)	No 12 (31) Yes 27 (69)
Operation side; *n* (%)	Right 20 (51) Left 11 (28) Bilateral 8 (21)
Levels of neck dissection; Level included; *n* (%)	LII 29 (74) LIII 36 (92) LIV 36 (92) LV 26 (67) LVI 35 (90)
Complications; *n* (%)	No 22 (56) Yes 17 (44)

*n* number, *L* level

By the time, PTC patients were sent to our department because of lateral LNM, and US had been performed already, thus limiting the number of preoperative US at our institution. The suspicious levels were unspecified in the US reports, because at our department, the aim of preoperative US is not to clarify the levels of LNMs. Thus, it was impossible to retrospectively evaluate the number and levels of lymph nodes from US images, and comparison between US and MRI on these parameters was inhibited.

Study population was young with a mean age of 45.5 years. The majority of patients were otherwise healthy, since 32 (82%) had ASA score 1–2 (Classification by American Association of Anesthesiologists) and CCI score 0–1 (Charlson Comorbidity Index).

We compared the levels of LNMs in MRI with the histopathology of the lateral ND preparate level-by-level. All patients who had undergone lateral ND showed nodal metastases on histopathology. Because Levels III and IV are most commonly affected and basically always included in ND in these patients (in 97% of patients in this cohort), we focused on Levels II and V. As NDs were performed on levels with negative preoperative MRI findings (29 out of all 64 dissected levels, in 16% of Level II NDs and in 75% of Level V NDs), we had an opportunity to study both the true and false positives but also the true- and false-negative results of MRI (Table 2). Regarding Level II, there were 12 (38%) patients with false-positive scans and one (3%) patient with false-negative scan. Regarding Level V, there were two (6%) patients with false-positive scans and three (9%) patients with false-negative scans. The three patients with false-negative MRIs for Level V presented with large bulky lateral LNMs. Their ND included Level V in all cases irrespective of the scan report. Furthermore, MRI seemed inadequate to assess ENE. Histopathology revealed ENE in 24 (62%) patients, although MRI indicated it only in eight patients (21%; *p* = 0.39).

**Table 2:** Correlation of preoperative MRI with histopathology to show the number of individual levels with pathological lymph nodes on the lateral neck in Level II (A), Level V (B), and the statistical correlates for MRI (C)

MRI positive	Histopathology malignant		
	Yes	No	Total
A. Level II			
Yes	15	12	27
No	1^a^	3	4
Total	16	15	
B. Level V			
Yes	6	2	8
No	3^a^	20	23
Total	9	22	

%	Level II (95% CI)	Level V (95% CI)
C. Accuracy of MRI		
Sensitivity	94 (70–100)	67 (30–93)
Specificity	20 (4–48)	91 (71–100)
PPV	56 (35–75)	75 (35–97)
NPV	75 (19–99)	87 (66–97)

*PPV* positive predictive value, *NPV* negative predictive value, *CI* confidence interval

^a^One patient excluded because of indefinite histopathology

Seventeen patients experienced 18 postoperative complications. These were categorized according to Dindo et al. [15]. The most common postoperative complication was hypocalcemia (Grade I), seen in six patients (15%). Four patients (10%) experienced postoperative weakness of the accessory nerve (Grade Id). Neural function recovered completely in two patients in a median time of 5 months but persisted in two (5%). Three patients (7%) developed Horner’s syndrome (Grade Id). One patient (3%) had acute postoperative arterial bleeding (Grade IIIb), which required immediate re-operation. One patient (3%) developed chylus leak (Grade IIIb), which was managed operatively twice. One patient (3%) developed a postoperative seroma (Grade I), which required puncture. Two (5%) postoperative infections (Grade II) were treated with antibiotics. The follow-up time varied from 8 to 32 months (median, 13 months).

## Discussion

The objective of the present study was to evaluate the ability of preoperative MRI to assess the distribution of lateral LNMs in patients with regionally metastatic PTC. MRI was fairly specific in diagnosing lateral LNMs for Level V, with a high negative predictive value. For Level II, MRI was highly sensitive but included many false positives in the present study. Occult metastases were more common at Level II (25%) than at Level V (13%).

We have regional management algorithm for patients with PTC. Patients are primarily treated at the Department of Surgery. At our institution, we perform surgery for all patients with PTC with lateral neck metastases. US together with fine needle aspiration biopsy are used to diagnose the disease, preoperative MRI; however, we use for staging and surgical planning. Unlike US studies, dedicated head and neck radiologist performs most MRI studies at our institution, possibly also contributing to the results and highlighting the accuracy of our MRI reports.

According to the current management guidelines, CT and MRI are used as an adjunct to US for patients with PTC with clinical suspicion for advanced disease, including invasive primary tumor, or clinically apparent multiple or bulky lymph node involvement [16]. Although iodine containing contrast agent should gradually disappear within 2 months, we favor MRI over CT at our institution to show possible lateral LNMs [16]. PTC patients in the present study were young, as also others have pointed out, although the mean age has slightly risen over the years [17]. Thus, MRI seems a more preferred method to avoid radiation dose in this patient population. In some patients, FDG-PET–CT may show additional value in evaluating regional or distant metastatic disease or recurrences, but its routine preoperative use is not recommended [18, 19].

We feel that our current MRI protocol (axial fat-saturated T2-weighted, axial T1-weighted, axial fat-saturated T1-weighted, axial and coronal gadolinium-enhanced, fat-saturated T1-weighted images) is sufficient and provides good quality for evaluation of the neck. Including pre-gadolinium T1 fat-suppressed sequence allows identifying very small nodes containing thyroglobulin, which may be otherwise overlooked. Although radiologists commonly opt out, reporting the suspected levels on MRIs may help the surgeon to guide the extent of ND, especially when considering omitting surgery of inconclusive levels.

Prophylactic lateral ND is not generally recommended for patients with PTC [20, 21]. The extent of ND in regionally metastatic PTC remains unsettled due to the lack of prospective studies, but as “berry picking” increases the risk for recurrences, an agreement lies on some type of ND as the treatment of choice for LNM [14, 22–27]. Since LNMs are most commonly found at Levels III and IV, including these levels in ND has been recommended in every patient with suspected lateral LNM [27]. Others prefer including also Levels II and V because of the lack of sensitivity of preoperative imaging to define these areas [3]. On the contrary, some rely on preoperative imaging to guide the extent of ND with the intention to avoid complications related to dissecting Levels II B and V [8]. The extent of ND and related possible complications requires careful consideration due to the reverse risks of surgical and oncological morbidity. Although patients with PTC are generally young with good overall survival, the risk for locoregional recurrences is known to be higher in patients with lateral LNMs [8]. Whether MRI can provide additional help in determining the extent of ND remains unsettled. Therefore, we aimed to assess both the rates of false-positive and false-negative findings in MRI, concentrating on the most discordant Levels II and V.

In the present study, ND included Levels II and especially V more often than the preoperative MRI indicated. This is an advantage, as previous studies usually lack information about the false-negative rate of imaging to assess neck metastases in PTC [28]. In our material, both clinical and pathological lateral LNMs were more common at Level II than at Level V. The limited number of patients undoubtedly affects the statistical results (Table 2). The ability of MRI to correctly indicate the presence of pathological lymph nodes was 56% for Level II and 75% for Level V. Likewise, the negative predictive value was higher for Level V (87%) than for Level II (75%). MRI’s sensitivity was 94% in Level II compared with 67% in Level V. This difference is probably attributable to the criteria for interpreting the imaging reports: the three patients with false-negative MRI scans for Level V had bulky nodal mass on the lateral neck, but the largest volume was categorized to be located either at Level III or at Level IV on imaging, and were thus regarded as nodes of either of these levels. As for Level II, 12 false-positive findings led to relatively low specificity (20%) and positive predictive value (56%) of MRI. Based on these findings, although it seems putatively appropriate to utilize preoperative MRI to omit Level V ND, this needs to be confirmed in larger cohorts before changing general practice and guidelines. On the contrary, using preoperative MRI to assess the probable inclusion/exclusion of Level II in ND seems unjustifiable, as we had a fairly high number of false-positive cases at this level. In our cohort, however, Level II was not subdivided into A and B in all cases in the pathological analysis. Still, routine dissection of Level II B would most likely be exaggeration and increase complications.

We showed that ENE was common and diagnosed after ND in 62% of patients, similar to that published by Lee et al. [29], but preoperative MRI indicated it only in 33% of them. In squamous cell carcinoma (SCC) of the head and neck, MRI seems to underestimate the occurrence of ENE [30, 31]. Steinkamp et al. reported sensitivity of 74% and specificity of 72% for MRI in detecting ENE, while the false-negative rate was 33% in the study by Shaw et al. [30, 31]. Similar to SCC [32], ENE is an independent predictor for locoregional recurrence in PTC [33–35] even in patients less than 45 years of age [36], but shows no clear impact on disease specific survival [35]. The follow-up time in the present study was too short to make conclusions about survival, but based on the literature, it seems that ENE in PTC does not compromise overall survival in contradiction to SCC [32] of the head and neck.

Our complication rate was 44%, of which only 5% represented Grades III–IV [15]. Including Levels II B and V in ND increases the risk for accessory nerve injury [22], which can cause additional discomfort and diminished quality of life [37]. In our cohort, spinal accessory nerve dysfunction occurred in the three patients with bulky lateral neck mass and with clinically negative MRI scans for Level V, and in one patient with clinically positive nodes at Level V. In these patients, ND of Level V was inevitable. Neural dysfunction remained in two (5%) of them, which is in line with that presented by Glenn et al. [38]. Thus, based on our limited number of patients, ND of Level V increases the risk for neuropathic morbidity in patients with lateral LNMs, and omitting this level would be most desirable when oncologically possible. Horner’s syndrome developed in three patients (7%), which is comparable to the literature [39–41].

This study presents with limitations due to the retrospective design. The number of patients was limited to draw definite conclusions on omitting ND on specific levels. In addition, patients had only a short follow-up time. The re-evaluation of all preoperative MRIs to standardize the scans may be regarded as strength in this study. Only comparable MRI sequences were included in this study.

## Conclusions

In this patient cohort, preoperative MRI was fairly accurate to assess the distribution of positive LNMs on the lateral neck—especially regarding Level V—in patients with PTC, although the ability to show ENE was low. It seems that preoperative MRI may help to perform a more targeted lateral ND in the primary treatment setting, although the number of patients in the present study was too limited to draw any definite conclusions on possibly omitting ND on specific levels. MRI showed more false positives and more occult metastases at Level II than at Level V, and in general, Level V was less often involved with LNMs. We suggest conducting imaging studies in the future with more patient data to accomplish targeted surgical fields and minimal surgical morbidity in this patient group.
